# Global Identification of HIF-1α Target Genes in Benzene Poisoning Mouse Bone Marrow Cells

**DOI:** 10.3390/ijerph15112531

**Published:** 2018-11-12

**Authors:** Zhaodi Man, Xing Meng, Fengxia Sun, Yunqiu Pu, Kai Xu, Rongli Sun, Juan Zhang, Lihong Yin, Yuepu Pu

**Affiliations:** Key Laboratory of Environmental Medicine Engineering, Ministry of Education of China, School of Public Health, Southeast University, Nanjing 210009, China; 220162973@seu.edu.cn (Z.M.); dzmx925@126.com (X.M.); 220152915@seu.edu.cn (F.S.); 230149559@seu.edu.cn (Y.P.); 230189311@seu.edu.cn (K.X.); 101012172@seu.edu.cn (R.S.); 101011288@seu.edu.cn (J.Z.); lhyin@seu.edu.cn (L.Y.)

**Keywords:** benzene, HIF-1α, hematopoietic toxicity, ChIP-Seq

## Abstract

Benzene is a hematopoietic toxicant, and hematopoietic cells in bone marrow (BM) are one of the main targets for its action, especially hematopoietic stem cells (HSCs). Hypoxia-inducible factor-1α (HIF-1α) is associated with the metabolism and physiological functions of HSCs. We previously found that the mechanism of regulation of HIF-1α is involved in benzene-induced hematopoietic toxicity. In this study, chromatin immunoprecipitation sequencing (ChIP-Seq) technologies were used to analyze the genome-wide binding spectrum of HIF-1α in mouse BM cells, and specific HIF-1α target genes and pathways associated with benzene toxicity were screened and validated. By application of the ChIP-Seq technique, we identified target genes HIF-1α directly binds to and regulates. Forty-two differentially down-regulated genes containing the HIF-1α specific binding site hypoxia response element (HRE) were found, of which 25 genes were with biological function. Moreover, the enrichment analysis of signal pathways indicated that these genes were significantly enriched in the Jak-STAT signaling pathway, Natural killer cell mediated cytotoxicity, the Fc epsilon RI signaling pathway, Pyrimidine metabolism, the T cell receptor signaling pathway, and Transcriptional misregulation in cancer. After verification, 11 genes involved in HSC self-renewal, cell cycle, differentiation, and apoptosis pathways were found to be significantly reduced, and may participate in benzene-induced hematotoxicity. Our study provides a new academic clue for the mechanism of benzene hematotoxicity.

## 1. Introduction

Benzene, a ubiquitous environmental pollutant and a recognized human carcinogen [1,2,3,4], easily enters the human body through the respiratory tract and skin, which leads to chronic benzene poisoning and hematopoietic dysfunction. The main manifestations of its hematotoxicity are low bone marrow (BM) hyperplasia and peripheral blood cell count [5]. At present, it is believed that the toxicity of benzene is mainly caused by the metabolites of benzene. Benzene and its metabolites can cause hematopoietic toxicity through many mechanisms, such as oxidative damage, gene mutation, and epigenetic change [5,6]. The metabolites of benzene include phenol, hydroquinone, catechol, and 1,4-benzoquinone [7]. 1,4-benzoquinone can combine directly with biomolecules to produce reactive oxygen species (ROS), cause lipid peroxidation, and eventually result in acute oxidative damage [8,9,10,11]. 

Hematopoietic stem cells (HSCs) exist in hematopoietic niches. The hypoxic microenvironment helps to maintain their energetic metabolism mainly based on anaerobic glycolysis, which is necessary to keep their stemness and quiescence [12,13]. It is now well established that HIF-1α is highly expressed in long term proliferating HSCs [14]. Hypoxia inducing factors, especially cell signaling produced by HIF-1α, maintains the quiescence, survival, and metabolic phenotype of HSCs [15,16,17]. HIF-1α not only controls the microenvironment of HSCs through increasing anaerobic glycolysis, but also reduces the production of ROS by inhibiting the activity of nicotinamide adenine dinucleotide phosphate (NADPH) oxidases (NOXs) [18,19,20]. The hypoxic environment of HSCs is beneficial in reducing the generation of endogenous ROS [21,22]. 

We previously [23] found that the intracellular levels of ROS in benzene poisoning male C57BL/6 mice at different concentrations were increased with a significant fall in the expression of HIF-1α, indicating that HIF-1α was involved in damage in the mouse hematopoietic system induced by benzene. Hypoxia inducible factor-1 (HIF-1) is a heterodimeric transcription factor that consists of a constitutively expressed subunit, hypoxia-inducible factor-1β (HIF-1β), and a subunit regulated by the cellular O_2_ concentration, HIF-1α [23,24,25]. Under the condition of hypoxia, the pathway of ubiquitination and proteasomal degradation is blocked, which results in stable HIF-1α protein expression. Thus, HIF-1α combines with the β subunit (HIF-1β) to form HIF-1, then translocates into the nucleus. The transcriptional coactivator p300 (CBP/p300) binds to the hypoxia response element (HRE) sequence on the downstream gene to initiate or enhance the transcription of the effector gene [26,27,28]. Finally, many pathways are regulated, such as cell proliferation, apoptosis, angiogenesis, and erythrocyte maturation [29]. HRE is the DNA-binding site of HIF-1α. The target genes of HIF-1α, such as Vascular endothelial growth factor (VEGF), Erythropoietin (EPO) [30,31], phosphoglycerate kinase 1 (PGK1), Lactate dehydrogenase A (LDHA) [32], insulin-like growth factor (IGF) [33], and Bmi1 [34,35], have the site sequence on the HRE.

Regulation of HIF-1α is essential in bone marrow hematopoietic toxicity induced by benzene, but the specific mechanism has not been clarified. Chromatin immunoprecipitation sequencing (ChIP-Seq) is a high throughput method for detecting DNA histone modification in the whole genome by combining ChIP with sequencing technology. There are many HIF-1α target genes, and little is known whether these target genes are related to benzene hematotoxicity. In this study, ChIP-Seq was used to analyze the genome-wide binding spectrum of HIF-1α in mouse bone marrow cells. The purpose of this research is to explore specific HIF-1α downstream target genes and pathways by which HIF-1α participates in hematotoxicity caused by benzene.

## 2. Materials and Methods

### 2.1. Reagents

1,4-Benzoquinone and 37% formaldehyde solution were purchased from Sigma Co. (Sigma, St. Louis, MO, USA). Corn oil was obtained from COFCO (Beijing, China). The Chip Enzymatic Chromatin IP Kit was purchased from Univ Corp (CST, Danvers, MA, USA). The HIF-1α antibody was supplied by Univ Corp (NOVUS, Littleton, CO, USA). 

### 2.2. Animals, Treatments, and Blood Routine Examination

Male C57BL/6 mice aged 5–6 weeks (weighing 20 g ± 2 g) were purchased from the Animal Core Facility of Nanjing Medical University. Mice (*n* = 24) were randomly divided into three groups and exposed to benzene (vehicle: corn oil) at doses of 0 mg/kg body weight (b.w.) (Control, C), 150 mg/kg b.w. (Low Benzene, LB), and 300 mg/kg b.w. (High Benzene, HB) by subcutaneous injections for 15 consecutive days. The mice were killed by dislocation of cervical vertebrae. After 75% alcohol immersion for 5 min, the hind limbs of the mice were cut with scissors, and the skin and muscles were removed. The bone was cut off at both ends to expose the bone marrow cavity. Bone marrow cells were obtained by washing the bone marrow cavity with 1 mL of sterile phosphate buffered saline (PBS) using a syringe. The whole bone marrow cells were collected and fixed, then the HIF-1α antibody was used to capture the chromatin for later experiments. During the process of exposure, the general changes of mental status, behavioral activities, and food intake of the mice were observed daily. Blood samples were taken from eyeballs (EDTA anticoagulant). Hematological parameters, such as white blood cells (WBC), red blood cells (RBC), and platelets (Plt), were measured by an automatic blood cell counting instrument. This study has been approved by the Research Ethics Committee of the Southeast University (approval number: 20140618).

### 2.3. Chromatin Immunoprecipitation (ChIP) Assay

Approximately 4 × 10^6^ BM cells were required per experiment. Briefly, cells were cross-linked with 0.54 mL 37% formaldehyde that was added to the minimal cell culture medium and mixed thoroughly for 10 min at room temperature, and the crosslinking was then quenched with 2 mL 10× glycine for 5 min. Cross-linked cells were washed twice with ice-cold PBS, and resuspended in 1 mL ice-cold lysis buffer with 5 μL protease inhibitor cocktail (PIC) and 5 μL phenyl-methylsulfonyl fluoride (PMSF). Cells were then resuspended and incubated on ice for 30 min. Subsequently, cell lysate was sonicated to shear chromatin to an average length of 150–900 base pairs (bp) and centrifuged for 10 min at 15,000 rpm in a 4 °C microcentrifuge. To each reaction tube, 10 μg sheared chromatin DNA was added, followed by 1 × ChIP buffer to a total volume of 500 μL, and then it was mixed on ice. Ten microliters of the samples were absorbed from each tube as 2% samples for input control (Input). Ten micrograms of chromatin was immunoprecipitated overnight at 4 °C with 2 μL of HIF-1α antibody (NOVUS), and 2 μL of control IgG antibody (Cell Signaling) or 10 μL of negative control protein H3 antibody (Cell Signaling). Thirty microliter Protein G Magnetic Beads (Millipore) were added and incubated for 2 h at 4 °C. Beads were successively washed with 1 mL of different buffers: Low-salt immune complex wash buffer (300 μL 10× ChIP buffer, 2.7 mL water) and high-salt immune complex wash buffer (100 μL 10× ChIP buffer, 900 μL water). Fifty microliters of the reverse cross-linking buffer was added to the eluted chromatin and the supernatant was then transferred. Finally, all tubes were incubated with 6 μL of 5 mol/L NaCl and 2 μL of proteinase K at 65 °C for 2 h. The DNA was purified by centrifuge column. The DNA from ChIP was quantified via Quant IT fluorescence assay (Life Technologies, Waltham, MA, USA). Illumina sequencing libraries were generated with the NEBNext^®^ Ultra™ DNA Library Prep Kit (New England Biolabs, Ipswich, MA, USA) following the manufacturer’s manual. The library quality was determined by using the Agilent 2100 Bioanalyzer (Agilent, Santa Clara, CA, USA), and then subjected to high-throughput 150 bp-end sequencing on a Illumina Hiseq sequencer.

### 2.4. Data Analysis

The genes corresponding to the differentially enriched peak located in the promoter region were selected by reference to the specific binding site HRE (aCGTG/gCGTG) of HIF-1α. Then, based on the location of the corresponding chromosomal sequences of each gene, the Mouse GRCm38/mm10 database of UCSC was used to find out whether there was a gene sequence corresponding to the differential enrichment peak in these gene sequences. If the HRE site of HIF-1α was found in this sequence, it was initially judged to be a positive gene. Afterwards, the positive genes that were initially screened in the previous step were searched in the Pubmed database for the biological function (gene ontology (GO) biological function). And the genes with clear function were again considered as positive genes.

### 2.5. Real-Time PCR

The screened differentially expressed genes were verified by RT-PCR. The reaction system was 20 μL (Table 1). Primers are shown in Table 2. After RNA extraction, reverse transcription of RNA to cDNA was performed with PrimeScript TM RT Master Mix. The program thermocycler of PCR was performed as follows: The program was started with an initial melt step at 95 °C for 30 s, then 95 °C for 5 s, 60 °C for 34 s, and run for 40 cycles. The *β*-actin was taken as the internal reference gene, and the relative expression level of genes was calculated by the 2^−ΔΔ*C*(t)^ method. The concentration of cDNA we used in the study was 500 ng/μL.

### 2.6. Statistical Analysis

Differences among multiple groups were analyzed by one-way analysis of variance (ANOVA) using SPSS 16.0 software (SPSS, Chicago, IL, USA). The comparison between the two groups was conducted by the least significant difference (LSD) test. All data was expressed by mean plus and minus standard deviation. The *p* values < 0.05 were considered statistically significant.

## 3. Results

### 3.1. Benzene Induced Hematopoietic Toxicity and Increased ROS in Mice

Our previous study [23] demonstrated that red blood cells (RBC), white blood cells (WBC), hemoglobin (Hgb), and platelets (Plt) decreased significantly in 150 mg/kg (LB) and 300 mg/kg (HB) groups, along with a fall in the proportion of HSCs (Control, 0.12%; LB, 0.03%; HB, 0.03%; *p* < 0.05) [23]. There was a distinct increase in the ROS level at a dose of 150 mg/kg. Therefore, the following chip samples of the benzene exposure group were selected for the LB group. Finally, according to the results of blood routine, two samples with obvious differences from the LB group and two samples from the control group were selected (L1, L2, C1, C2).

### 3.2. ChIP-Seq Analysis

**Figure 1:**
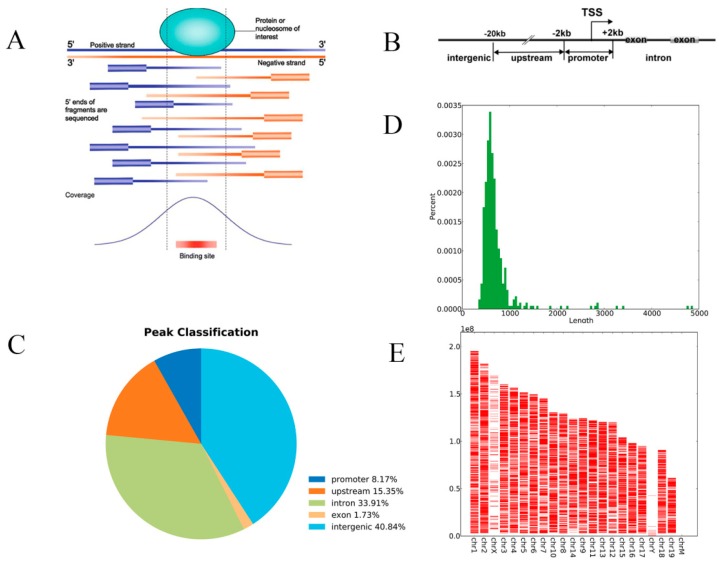
The results of chromatin immunoprecipitation sequencing (ChIP-Seq). Two samples from the low benzene (LB) group and two samples from the control group were selected. (**A**) Enriched peak. (**B**) Peak annotation. The enriched peaks were identified and classified as follows: Promoter peaks, upstream peaks, intron peaks, exon peaks, and intergenic peaks. (**C**) Peaks classification. Of the total generated significant peaks in the control sample (*p* < 0.0001), 8.17% were within the promoter region, 33.9% were within introns, and 40.84% were within the intergenic region. (**D**) Peak length distribution. The length distribution of the enrichment peak is calculated to infer the length of the DNA binding region. (**E**) ChIP-Seq peaks over chromosomes. The distribution of peaks in the genome was statistically enriched, and the chromosomes in the DNA binding region were viewed as a whole.

The DNA fragments were randomly fragmented by sonication treatment, and the sequenced DNA fragments terminal was mapped at different positions on the genome. Assuming each fragment was 50 in the 200~300 bp region, it was extended and the resulting signal map (“coverage”) was visualized as a typical peak shape. The actual DNA sequence combined by HIF-1α should be located at the maximum point (bottom) of the coverage map; that is, an enriched peak (Figure 1A). After sequencing, image recognition, base calling, and quality filtering, high quality reads (Raw Data) were generated from the Illumina sequencer (Table 3). Then, trimming the joint sequence, the trimmed reads were aligned to the mouse reference genome (UCSC MM10) for peak calling. By comparing the enriched peak with the mouse genome in the UCSC RefSeq database, the enriched peaks were divided into promoter peaks, upstream peaks, intron peaks, exon peaks, and intergenic peaks (Figure 1B,C). Then the differentially enriched peaks (DEPs) were further identified by diffReps software and annotated with the latest UCSC RefSeq database to connect the peak information with the gene annotation. Taking advantage of the promoter region (transcriptional start site, TSS–2000~TSS+2000) on the genes corresponding to the enriched peaks, we performed GO functional analysis and Kyoto Encyclopedia of Genes and Genomes (KEGG) pathway analysis to annotate, and speculated the biological functions and pathways that these peaks may participate in. 

**Figure 2:**
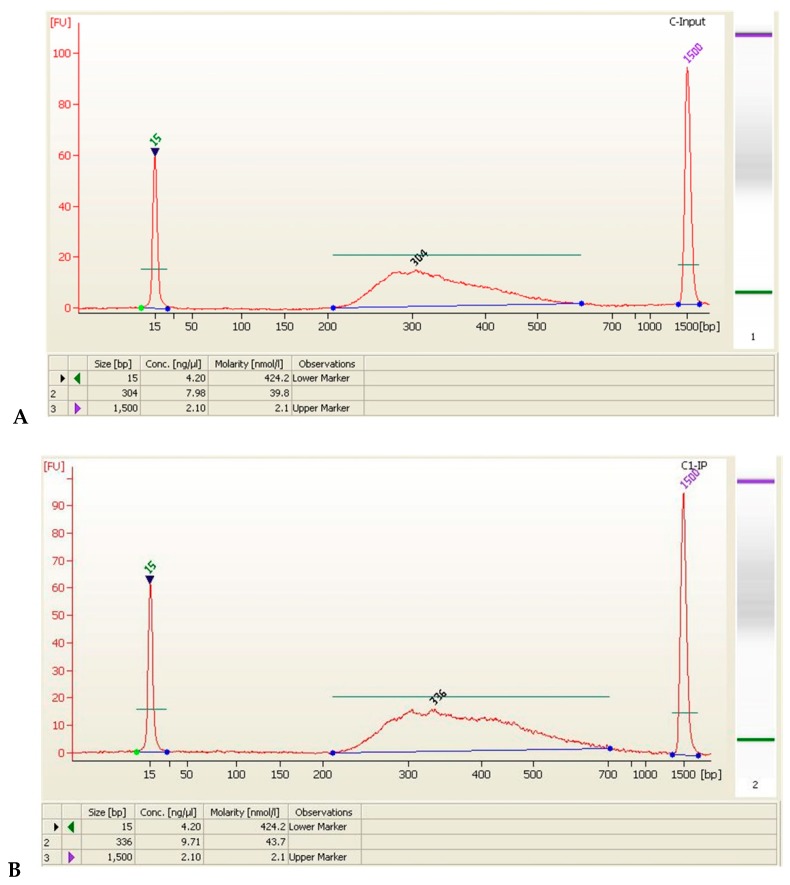

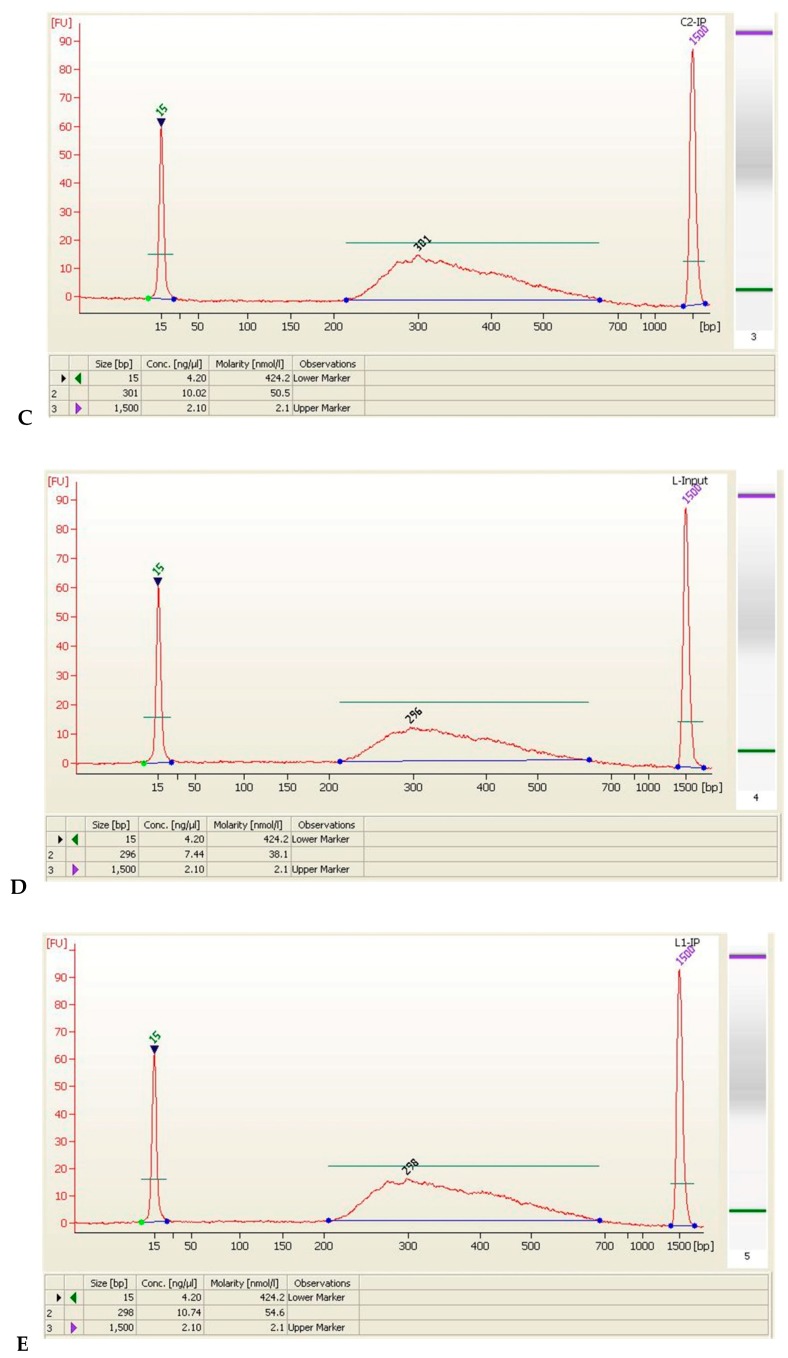

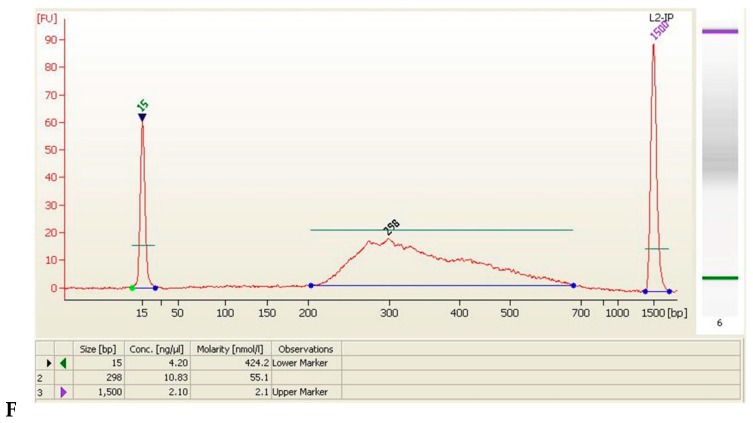
Quality evaluation of control and LB groups by sequencing library. Two samples from the LB group and two samples from the control group were selected (L1, L2, C1, C2). (**A**) C-Input; (**B**) C1-IP; (**C**) C2-IP; (**D**) L-Input; (**E**) L1-IP; (**F**) L2-IP.

To gain further insight into biological pathways associated with the significantly up or down-regulated genes identified by HIF-1α, analysis using the Database for Annotation, Visualization, and Data quality control (QC) bioinformatics tool was performed (Table 4, Figure 2). 

### 3.3. Screening of HIF-1α Responsive Genes

The reduced differential enrichment peak was located in the promoter region, and then the peak was matched to the corresponding gene. A total of 353 up-regulated and 245 down-regulated enrichment peaks in the promoter region were found in the LB group. The top 15 significantly up-regulated or down-regulated genes were chosen and are shown in Table 5. Among the 245 down-regulated genes, 42 genes containing the HIF-1α specific binding site HRE (aCGTG/gCGTG) in the gene sequence of the differentially enriched region were selected using the UCSC database.

Therefore, we preliminarily confirmed that 42 response genes were the target gene of HIF-1α. By searching for the function in the Pubmed database, we found that there were 25 genes involved in the following functions: Promoting cell proliferation, migration, invasion, growth, and metastasis. Thus, these genes were selected for further validation.

### 3.4. Gene Ontology (GO) Analysis

**Figure 3:**
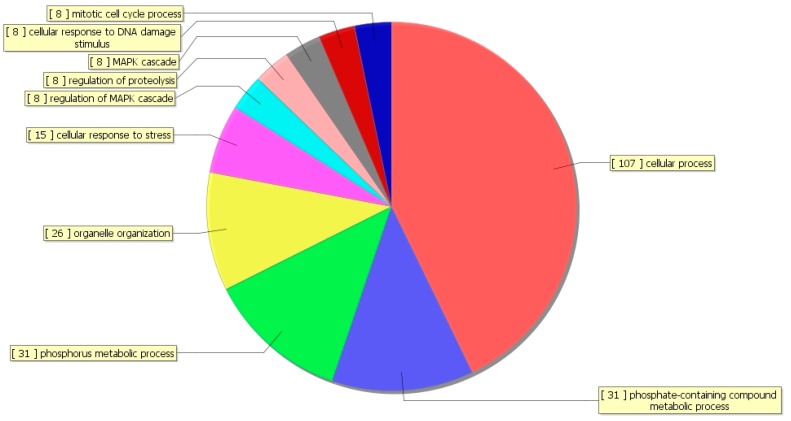
Gene ontology (GO) biological process classification. The pie chart shows the GO biological process (GOBP) sequencing result of genes corresponding to the reduced enrichment peak in the 150 mg/kg (LB) group compared to the control group, with the top ten counts of the significant enrichment terms.

The ontology covers three domains: Biological Process (BP), Cellular Component (CC), and Molecular Function (MF). It is interesting to note that GOBP terms were related to the cellular process, phosphorus metabolic process, phosphate-containing compound metabolic process, cellular response to stress, and MAPK cascade (Figure 3).

### 3.5. Kyoto Encyclopedia of Genes and Genomes (KEGG) Analysis

**Figure 4:**
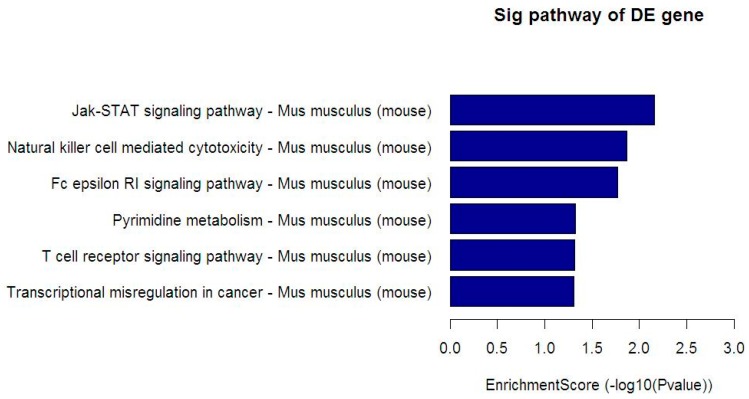
Kyoto Encyclopedia of Genes and Genomes (KEGG) pathway associated with HIF-1α responsive genes. Through bioinformatics analysis, multiple pathways of HIF-1α differentially binding target genes and HIF-1α coregulated genes were obtained in mice bone marrow (BM) cells compared with the control group. The HIF-1α target genes, which correspond to the reduced enrichment peak in the 150 mg/kg (LB) group, were significantly enriched in the KEGG signaling pathway.

The genes corresponding to the differentially enriched peaks in the promoter region (TSS–2000~TSS+2000) were used to analyze the pathway, in order to elucidate and speculate the pathways in which these enriched peaks (proteins) may be involved. Similarly, Figure 4 displays the KEGG pathway, which was significantly enriched in the HIF-1α responsive gene corresponding to the reduced enrichment peaks expressed in the LB benzene treatment group compared with the control group, including the Jak-STAT signaling pathway, Natural killer cell mediated cytotoxicity, Fc epsilon RI signaling pathway, Pyrimidine metabolis, T cell receptor signaling pathway, and Transcriptional misregulation in cancer. The actually matched HIF-1α responsive gene count and name are displayed in Table 6.

### 3.6. Validation of HIF-1α Target Gene

The mRNA level of HIF-1α target genes was detected in BM cells of C57BL/6 mice between the benzene exposure and control groups by RT-PCR. Then the results of RT-PCR and ChIP-Seq were analyzed together to screen out candidate critical genes. The mRNA level of 11 out of 25 genes was down-regulated in BM cells in 150 mg/kg (LB) and 300 mg/kg (HB) benzene-exposed mice compared with the control group (Table 7). 

## 4. Discussion

With the wide application of benzene in coatings, dyes, spray paint, and other industries, occupational benzene exposure and its health hazards are important public health issues [36]. Elucidation of the mechanism of benzene toxicity can provide a scientific basis for finding the early damage markers, and is helpful for prevention and treatment strategies for benzene poisoning. 

Benzene enters into the human body mainly through respiratory inhalation and skin contact [4]. Part of it is excreted from the body and part metabolized in the liver and BM. Firstly, phenol and catecholamine are produced from benzene under the action of cytochrome oxidase in the liver [7]. Secondly, these compounds are catalyzed by myeloperoxidase (MPO) in BM to be the ultimate carcinogen, benzoquinone. BM is an important target organ of benzene toxicity. Therefore, the BM cells of benzene-exposed mice were selected for ChIP-Seq. ROS levels in quiescent stem cells are kept low, thus supporting their ability for long-term repopulation. Survival in this low-oxygen microenvironment requires significant metabolic adaptation [37]. Hypoxia inducible factors (HIFs) are the most direct regulatory factor in the hypoxic environment [38,39,40], which can regulate oxidative phosphorylation and glycolysis transformation. In the bone marrow microenvironment, the HIF family is involved in glycolytic metabolism, thereby providing sufficient amounts of ATP to maintain HSC activity.

We firstly reported the genome-wide study of HIF-1α target genes in benzene-exposed mice. Compared with the control group, there were 42 differentially enriched genes in the benzene poisoning group. Through analyzing the GO function and signal pathway of differentially expressed genes, we found that these genes were mainly enriched in signal pathways related to cell apoptosis, differentiation, proliferation, oxidative stress, and the hematopoietic system. In addition, our early results showed that exposure to a certain dose of benzene could induce HIF-1α degradation, and decrease the proportion of hematopoietic stem cells and number of peripheral blood cells [23]. Thus, the abnormal expression of HIF-1α and its downstream genes may be involved in benzene-induced hematotoxicity. 

The GO function of differentially enriched genes and the RT-PCR analysis of mouse bone marrow cells showed that 11 genes were down-regulated in the benzene exposure group compared with the control group. Among them, ipo4 [41] functions as a transporter of HIF-1α. Ribosomal protein S3a1 (Rps3a1) [42], calpain 5 (Capn5) [43], skeletal muscle α-actin 1-encoding gene (Acta1), and ganzyme B (Gzmb) [44] are all related to cell apoptosis and differentiation. The up-regulation of protein tyrosine phosphatase 4A3 (Ptp4a3) [45] activates Src kinase, which promotes many cell signaling pathways, and cell proliferation, growth, and migration. Research has shown that RNA-binding motif protein 45 (RBM45) transfers from nucleus to cytoplasm under oxidative stress, and this redistribution regulates Kelch-like ECH-associated protein 1 (KEAP1) protein levels, thereby inhibiting NF-E2-related factor 2 (NRF2) activity and reducing cellular antioxidant responses. However, activation of NRF2 may temporarily protect HSCs from the harmful effects of ROS. Once the ROS level recovers, it promotes the long-term decline of HSC self-renewal [46]. Elevating ROS levels rise and lead to cell death [47], which is of great importance in cancer therapy strategies. It has been reported that the Rab GTPase activating protein 1-Like (Rabgap1l)-mediated tyrosine kinase signal transduction pathway plays a major role in the pathogenesis of leukemia [15]. The role and mechanism of specific HIF-1α response genes deserves further study.

Here, the ChIP-Seq technique was used to study the downstream specific genes and signaling pathways regulated by HIF-1α in benzene-induced hematotoxicity, and to explore the role and molecular mechanism of the ROS mediated HIF-1α pathway in hematotoxicity caused by benzene. Our results demonstrate that HIF-1α coregulated with its target genes and pathways in coping with benzene-induced hematopoietic damage. Further research is needed to find the interaction of HIF-1α target genes in the hematopoietic related signaling pathway. Our study provides new clues for screening critical HIF-1α responsive genes and elucidating the mechanism of benzene hematotoxicity.

## 5. Conclusions

Benzene exposure may destroy the bone marrow microenvironment and decrease the expression of HIF-1α, indicating that HIF-1α plays an important role in benzene-induced hematopoietic damage. It is well known that there are many HIF-1α target genes, but which of them participate in benzene toxicity remains unclear. In the present study, the HIF-1α target genes related to benzene toxicity were screened by the ChIP-Seq technique, and we further identified differential HIF-1α responsive genes and pathways following benzene exposure. Eleven HIF-1α down-regulated genes were validated as critical genes, which were involved in HSC self-renewal, cell cycle, differentiation, and apoptosis pathways. This study provides new insight into the mechanism of benzene-induced hematopoietic toxicity.

## Figures and Tables

**Table 1:** RT-PCR mixture.

Reagent	Volume
DNase/RNase-Free Water	6 μL
SYBR Green PCR Master mix (Toyobo)	10 μL
Forward /Reverse primer (100 μM)	0.8 μL
ROX Reference Dye	0.4 μL
cDNA	2 μL

**Table 2:** PCR primer sequences of HIF-1α target genes.

Gene	Forward Primer	Reverse Primer
*β*-actin	CTATGCTCTCCCTCACGCCA	TCACGCACGATTTCCCTCTC
Ptp4a3	CCTGTAAGGCAGCCCCAACTA	GTGTCTTAGCCAGGGTTTTATG
Samd4	CAGACGAGGAAGAGTAGAGGG	ACAGACGCATTACTATCACCAA
Ifitm3	GAGGACCAAGGTGCTGATGTT	TAGCCTATGCCTACTCCGTGAA
Gzmb	GCCAGTCTTTGCAGTCCTTTA	CTCTGATTACCCATCGTCCCT
Acta1	CCTTCTGACCCATACCTACCAT	AAGCCTCACTTCCTACCCTCG
Rbm45	TTTAGGTTCAGCCAAGAGTGC	CGGGAGAAGTTCAAGGTGTAT
Capn5	TGATTCCTCTTAGCCTCGTCA	GTGGATTTCACAGGTGGTGTT
Rps3a1	AGCAAGGCTCACTTCAAACAC	TTAGGAACATCGGGAAGACAC
Ipo4	AGCCACTCCTCCATGTCTTCC	CATCTTTGGGTTGGGCGTACT
Asb15	GAGCCTCAGCATAATCTCATC	TATACTTCGCCGTCTCCAATA
Rabgap1l	AGAGGCGGCTTAGTTGTTTGG	GCGGTCTACCTGTTGATTGCC

**Table 3:** Sequencing reads statistics of the ChIP experiment.

Name	Raw Reads	Mapped to Reference Genome	Mapped Percentage
C-Input	13,294,498	13,049,446	98.16%
C1-IP	16,759,284	16,585,336	98.96%
C2-IP	15,330,270	14,798,086	96.53%
L-Input	14,177,435	13,932,833	98.27%
L1-IP	17,959,618	17,666,744	98.37%
L2-IP	16,528,262	15,512,603	93.86%

**Table 4:** Quality control (QC) of the ChIP experiment.

Sample Name	Size (bp)	Concentration (ng/μL)	Concentration (nmol/L)	Volume (μL)	Total Amount (ng)
C-Input	304	7.98	39.8	20	159.6
C1-IP	336	9.71	43.7	20	194.2
C2-IP	301	10.02	50.5	20	200.4
L-Input	296	7.44	38.1	20	148.8
L1-IP	298	10.74	54.6	20	214.8
L2-IP	298	10.83	55.1	20	216.6

**Table 5:** Significantly changed genes corresponding to the differentially enriched peak.

Down-Regulated Gene Name	Fold Change	FDR	Up-Regulated Gene Name	Fold Change	FDR
Olfr1120	−138.5	0.000	Fpgt	128.8	0.005
Hilpda	−116.3	0.003	Lrriq3	128.8	0.005
Ebag9	−116.3	0.003	Pitpnm2	113.6	0.007
Ptp4a3	−105.4	0.002	Rspry1	111	0.006
Rgs1	−105.4	0.002	Fam192a	111	0.006
Tmc1	−99.9	0.005	Otud1	106	0.007
Snx33	−94.4	0.007	Rab5c	104.3	0.010
Spred3	−94.3	0.007	Edn3	102.3	0.004
Snhg17	−94.3	0.007	Ccdc88a	97.3	0.007
Olfr1113	−94.3	0.007	Sod2	96.7	0.012
Commd9	−89	0.005	Sept9	96.7	0.012
Rhno1	−89	0.005	C330013E15Rik	96.1	0.012
Foxm1	−89	0.005	Dynll1	95.5	0.012
Pgm5	−88.9	0.005	Gm13830	95.5	0.012
Mug1	−88.8	0.009	Asnsd1	94.1	0.007
Samd4	−88.8	0.009	4930486L24Rik	90.4	0.012

Note. FDR: The corrected p-value between control (0 mg/kg, C) and benzene exposure (150 mg/kg, LB) groups.

**Table 6:** Pathway Results.

Pathway	Total	Hits	Target Gene Name
Jak-STAT signaling pathway	155	5	CSF2RA, GRB2, PIK3CA, SPRED2, SPRED3
Natural killer cell mediated cytotoxicity	119	4	GRB2, GZMB, LCP2, PIK3CA
Fc epsilon RI signaling pathway	70	3	GRB2, LCP2, PIK3CA
Pyrimidine metabolism	104	3	CTPS, NT5C3B, TXNRD1
T cell receptor signaling pathway	105	3	GRB2, LCP2, PIK3CA
Transcriptional misregulation in cancer	178	4	BCL2A1C, GZMB, JMJD1C, LYL1

Note. Total represents the total count of the genes in the listed pathway. Hits is the actually matched enriched gene number in the pathway.

**Table 7:** Validation of differentially deregulated genes by RT-PCR.

Gene	2^−ΔΔ*C*(t)^	
	LB/C	HB/C
Ptp4a3	0.11985 ***	0.07554 ***
Samd4	0.10351 ***	0.04879 ***
Ifitm3	0.39707 *	0.53172
Gzmb	0.41548	0.35731 *
Acta1	0.06175 **	0.11185 *
Rbm45	0.70175	0.49878 **
Capn5	0.08935 ***	0.06172 ***
Rps3a1	0.48881 ***	0.57754 **
Ipo4	0.39123 **	0.31920 **
Asb15	0.09352 **	0.25600
Rabgap1l	0.23461 ***	0.41362 **

Note. Transcriptional level of HIF-1α target genes in mouse bone marrow cells. Eleven genes were validated in 150 mg/kg (LB) and 300 mg/kg (high benzene, HB) doses. * Significant difference compared to the control group, *p* < 0.05. ** Significant difference compared to the control group, *p* < 0.01. *** Significant difference compared to the control group, *p* < 0.001.
